# Mg-Doped PLA Composite as a Potential Material for Tissue Engineering—Synthesis, Characterization, and Additive Manufacturing

**DOI:** 10.3390/ma16196506

**Published:** 2023-09-30

**Authors:** Fawad Ali, Ans Al Rashid, Sumama Nuthana Kalva, Muammer Koç

**Affiliations:** Division of Sustainable Development, College of Science and Engineering, Hamad Bin Khalifa University, Qatar Foundation, Doha 34110, Qatar; anrashid@hbku.edu.qa (A.A.R.); sunu43717@hbku.edu.qa (S.N.K.); mkoc@hbku.edu.qa (M.K.)

**Keywords:** Additive Manufacturing, 3D printing, PLA/Mg composite, bone tissue engineering

## Abstract

Magnesium (Mg)/Polylactic acid (PLA) composites are promising materials for bone regeneration and tissue engineering applications. PLA is a biodegradable and biocompatible polymer that can be easily processed into various shapes and structures, such as scaffolds, films, and fibers, but has low biodegradability. Mg is a biocompatible metal that has been proven to have good biodegradability and osteoconductivity, which makes it suitable for bone tissue engineering. In this study, we prepared and characterized a Mg/PLA composite as a potential material for direct ink writing (DIW) in 3D printing. The results showed that the addition of Mg has a significant impact on PLA’s thermal and structural properties and has also significantly increased the degradation of PLA. XRD was used to determine the degree of crystallinity in the PLA/Mg composite, which provides insight into its thermal stability and degradation behavior. The crystallization temperature of PLA increased from 168 to 172 °C for a 15 wt% Mg incorporation, and the melting temperature reduced from 333 °C to 285 °C. The surface morphology and composition of these films were analyzed with SEM. The films with 5 wt% of Mg particles displayed the best-ordered honeycomb structure in their film form. Such structures are considered to affect the mechanical, biological and heat/mass transfer properties of the Mg/PLA composites and products. Finally, the composite ink was used as a feed for direct ink writing in 3D printing, and the preliminary 3D printing experiments were successful in resulting in dimensionally and structurally integral scaffold samples. The shape fidelity was not very good, and some research is needed to improve the rheological properties of the ink for DIW 3D printing.

## 1. Introduction

Polylactic acid (PLA) is a biodegradable and biocompatible polymer commonly used in tissue engineering and bone regeneration. It is a promising material for these applications because it can be easily processed into various shapes and structures, such as scaffolds, films, and fibers [1,2,3,4]. Additionally, PLA can be combined with other biomaterials, such as growth factors, to enhance its ability to support the growth and differentiation of cells. In tissue engineering and bone regeneration, PLA scaffolds are commonly used to provide a supportive structure for cells to attach and grow on [5]. These scaffolds can mimic the extracellular matrix of the tissue or organ being regenerated, which helps to promote the growth and organization of the cells [6,7]. Over time, the PLA scaffold will gradually degrade and replace the newly formed tissue or bone [8]. One of the critical advantages of PLA is its biodegradability, which means that the body can break it down over time without causing any adverse effects. This makes it a potentially valuable material for medical implants that must be gradually replaced as the body heals [8,9,10].

However, PLA is a relatively weak and brittle material [11], making it challenging to fabricate the complex and highly porous scaffolds needed for some tissue engineering applications. Additionally, PLA may not be the most suitable material for applications that require high mechanical strength, such as load-bearing bone replacements [11,12]. Various additives have been used to improve the mechanical properties of PLA for biomedical applications [13,14]. Another limitation of PLA is that its degradation rate can vary depending on the processing conditions and the presence of other materials, which makes it difficult to control the rate at which the scaffold degrades and is replaced by newly formed tissue or bone [15,16]. In some cases, the scaffold may degrade too quickly, leading to a loss of mechanical support for the cells before the tissue or bone fully regenerates. In other cases, the scaffold may not degrade quickly enough, resulting in scar tissue formation and the eventual failure of the tissue-engineered construct. Furthermore, these traditional biometals employed as temporary implants do not naturally break down within the body’s natural environment, potentially requiring a subsequent surgical procedure for removal once the healing process is complete [17]. Mg, on the other hand, is a naturally occurring element that is an essential component of bone tissue [18,19,20]. It has been shown to have several beneficial effects on bone cells, including promoting the growth and differentiation of osteoblasts (bone-forming cells) and inhibiting the activity of osteoclasts (cells that break down bone tissue) [21,22]. Its unique blend of strength, lightweight properties, and inherent biodegradability make magnesium (Mg)-based alloys a compelling choice for orthopedic implants and cardiovascular stents. Acting as metallic supports, Mg and its alloys, when used in scaffolds, offer essential mechanical reinforcement during the initial phases of tissue healing and cellular proliferation. Later on, their natural degradation and absorption by surrounding tissues obviate the need for additional surgical intervention for their removal [17].

Polylactic acid (PLA) and magnesium (Mg) are biocompatible materials that have been studied for bone regeneration and other biomedical applications. In particular, their combination into a composite material has shown promise for improving PLA’s mechanical properties and biocompatibility, making it more suitable for medical implants and other applications [22]. Mg-doped PLA composites have been synthesized by blending Mg with PLA or by incorporating Mg into the polymer chain during the synthesis of PLA. The Mg content, particle size, and distribution of Mg within the composites can affect their properties [23]. Mg-doped PLA composites have been shown to support the attachment, proliferation, and differentiation of various types of bone cells, such as osteoblasts and osteocytes, in in vitro studies [24]. The Mg content of the composites can affect their biocompatibility, mechanical properties, and proliferation. Mg-doped PLA composites have also been demonstrated to stimulate the formation of new bone tissue in animal studies.

This research aims to develop a PLA/Mg composite printable for a direct ink 3D printing technique for biomedical applications. DIW uses low temperatures, which can be beneficial for the layer-by-layer deposition of cells in the ink, which is otherwise not possible with a high-temperature manufacturing process. Direct ink printing is a 3D printing technique involving extruding material through a nozzle to build structures layer by layer. In this process, we produced some honeycomb structures with a PLA/Mg composite that can have multiple applications. Finally, we successfully 3D printed the PLA/Mg composite ink using a direct ink printing technique into sample scaffolds.

## 2. Materials and Methods

### 2.1. Materials

Polylactic acid (PLA) granules with a nominal size of 3–5 mm, a melting point of around 170 °C, and an average molecular weight of approximately 68,000 g/mol, were purchased from Goodfellow Cambridge Limited (Cambridge, England). Mg alloy (AZ61, purity 99.95%) powder with an average diameter of 100 μm was provided by Nanografi Nanotechnology (Ankara, Turkey). Chloroform (boiling point: 59.5–60.5 °C, density: 1.48 kg/L) was used as a PLA solvent supplied by VWR Chemicals, Darmstadt, Germany.

### 2.2. Solution Preparation and Thin Film Formation

PLA solution was prepared by mixing PLA granules in chloroform with a concentration of 20 g/L and mechanically stirring them for 2 h at 350 rpm. Varying amounts of Mg alloy powder (5 wt%, 10 wt%, 15 wt%, and 20 wt%, as reported in Table 1) were then added to the chloroform/PLA mixture solution for preparing PLA/Mg composites and, stirring was continued for 4 h at room temperature for the uniform distribution of Mg particles. The final mixture was poured onto a metal plate, left to dry for 24 h at room temperature, and peeled from the metal plate. After the chloroform evaporation, the PLA/Mg composites were acquired for characterization.

### 2.3. Characterization

A thermogravimetric analysis (TGA) (TA SDT 650) was used to determine the thermal stability of the PLA/Mg composites. These composites were taken into the ceramic crucible, and heated from room temperature to 500 °C with a heating speed of 10 °C/min under a nitrogen atmosphere. The cold crystallization temperature, melting point, and decomposition temperature of the differential scanning calorimetry (DSC) and the remaining mass percentage of the TGA at 500 °C were identified from the testing curves. The composite surface morphology was examined using a field emission scanning electron microscope functioning at a 2 kV accelerating voltage and a working distance of 9.0 mm. The composite composition was analyzed with energy-dispersive X-ray spectroscopy (FEI Quanta650FEG is used for imaging and Bruker Quantax400 for EDS). The X-ray diffractometer (XRD) was used to determine the crystal structures of the composites using Cu Kα radiation within an angle range of 10°–90° at a scan rate of 0.1 °/min. A Fourier transform infrared spectrometer (FTIR) was used to detect the functional groups in the PLA/Mg composites before and after decomposition.

### 2.4. Direct Ink Writing (DIW) 3D Printing of PLA/Mg Ink

**Figure 1:**
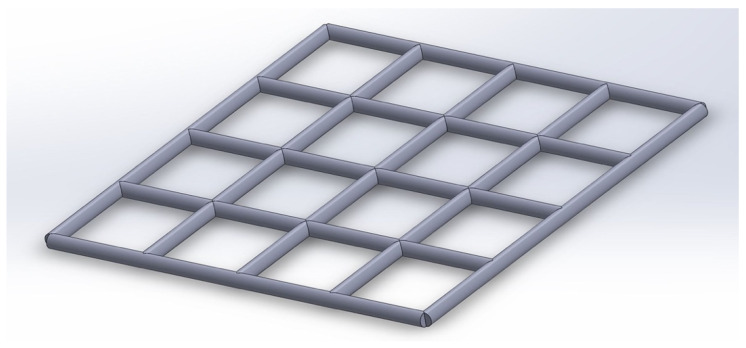
Designed mesh for the 3D bioprinting process.

The synthesized PLA/Mg composites were also used in the bioprinting process to investigate the feasibility of using the 3DP process for novel polymeric ink. A PLA/Mg composite with 5 wt% of Mg nanoparticles was used for DIW 3D printing. The viscosity of the PLA/Mg solution was determined using a viscometer (Brookfield DV2T, Middleboro, USA ), as controlled viscosity is vital for a successful 3D print. As reported in Figure 1, a simple mesh with overall dimensions of 12.4 mm × 12.4 mm × 0.4 mm was designed using Solidworks, with a strut thickness of 0.4 mm and spacing of 3 mm between the struts. The designed mesh was imported in STL format to Repetier-Host software for slicing and G-code generation. A TissueStart 3D bioprinter (from TissueLabs) was used to fabricate the designed part, with a printing speed of 2 mm/s, a layer height of 0.1 mm, and 100% infill. The fabricated structure was analyzed using a Zeiss microscope with 5× and 10× resolutions. A visual representation of the designed mesh along with the specifications and 3DP process parameters are reported in Figure 1 and Table 2.

## 3. Results and Discussion

### 3.1. Thermogravimetric Analysis (TGA)

**Figure 2:**
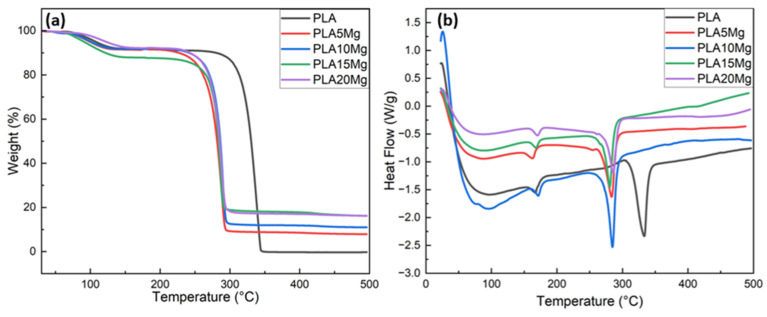
(**a**) TGA and (**b**) DSC results for PLA/Mg composites with varying amounts of Mg, showing the thermal stability and heat flow of PLA as the Mg content increases.

Thermogravimetric analysis (TGA) is a technique used to measure the weight change of a material as a function of temperature or time in a controlled atmosphere. TGA is commonly used to determine the thermal stability of a material, as well as its decomposition temperature and the weight loss resulting from the thermal decomposition. In the case of polylactic acid (PLA), TGA can be used to determine the temperature at which the material starts to degrade and the extent of degradation that occurs at different temperatures. TGA was used to assess the impact of Mg concentration on the thermal stability of PLA. According to Figure 2a, an initial mass loss was observed for all the samples after 70 °C, most likely because of the solvent evaporation. To ensure the removal of any residual solvent within the scaffolds, it is strongly advised to conduct a post-treatment involving a heat treatment at 40 °C. Pristine PLA degrades thermally between 300 °C and 350 °C, whereas PLA/Mg composites start to degrade sooner and do so between 250 °C and 300 °C. Mg addition speeds up heat breakdown in PLA. It is clear from these results that Mg incorporation speeds up the degradation of PLA when subjected to elevated temperatures. When the polymer is exposed to high temperatures, Mg acts as a catalyst for the PLA depolymerization reaction [25,26]. The residual amount of Mg shown at 500 °C agrees with the actual quantity used during the experiment. The minor value difference is likely derived from the handling of materials in such small amounts.

### 3.2. Differential Scanning Calorimetry (DSC)

**Figure 3:**
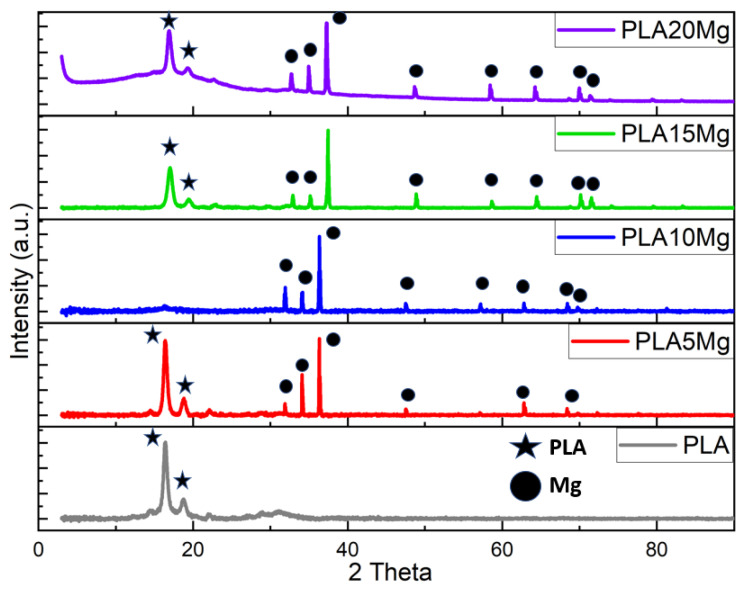
XRD peaks of PLA and PLA/Mg composites with various Mg amounts.

The thermal properties of PLA and PLA/Mg composites were studied using DSC, and the results are reported in Figure 2b. The characteristics of semi-crystalline PLA, i.e., the glass transition temperature (T_g_), melting temperature (T_m_), and decomposition temperature (T_d_), can be seen and compared in all the specimens, and the values are summarized in Table 3. It can be observed that the melting temperature of PLA increases with the addition of Mg particles. This can be attributed to the interaction of the polymer chains with the Mg particles, which reduces the mobility and lubrication of the PLA molecular chains. On the other hand, the addition of Mg suggests that the Mg particles act as a nucleation agent, which speeds up crystallization (i.e., the crystallization time decreases with the incorporation of Mg due to the heterogeneous nucleation effect of these particles) [27,28]. However, the degree of crystallinity does not change significantly as the intensity of the crystallization peaks remains unchanged with different Mg contents, as is evident from XRD peaks (Figure 3). The melting temperature for all the specimens was around 167 °C, with a slight increase with the addition of the Mg particles. PLA/Mg composites had higher melting temperatures than pure PLA, but there was a slight decrease in its melting temperature as the amount of Mg increased from 0 to 5 percent. Thus, it can be assumed that the crystalline structure of PLA is not much affected by the incorporation of Mg particles into the PLA matrix.

### 3.3. X-ray Diffraction (XRD)

Figure 3 shows the XRD spectra of the pure PLA and Mg-immersed samples. The Mg alloy peaks that appeared at 2θ = 16.5°, 34°, and 36° are intensified by increasing the Mg concentration in the PLA samples. The pure PLA exhibited a crystalline nature, and a peak appeared at 2θ = 16° and 19.5°, corresponding to the ‘α’ crystallization phase. Initially, increasing the Mg concentration increased the crystallinity of the PLA. This is because the Mg ions act as nucleating agents, promoting the formation of crystalline regions in the polymer matrix. As a result, the XRD pattern of the PLA with 5% Mg concentration showed more intense diffraction peaks, indicating a higher degree of crystallinity. Further, increasing the Mg concentration to 10% and 15% decreased the crystallinity of the PLA. This is because the Mg ions can disrupt the crystalline structure, leading to a more disordered arrangement of the polymer chains. As a result, the XRD pattern of the PLA with a 10% Mg concentration showed no peak, indicating a lower degree of crystallinity. The PLA peaks reappeared for 15 and 20% Mg concentrations with higher intensities. It is worth noting that the XRD peaks represent information on the degree of crystallinity of a very thin surface layer, and the bulk crystallinity might differ [29].

### 3.4. Fourier Transform Infrared Spectroscopy (FTIR)

**Figure 4:**
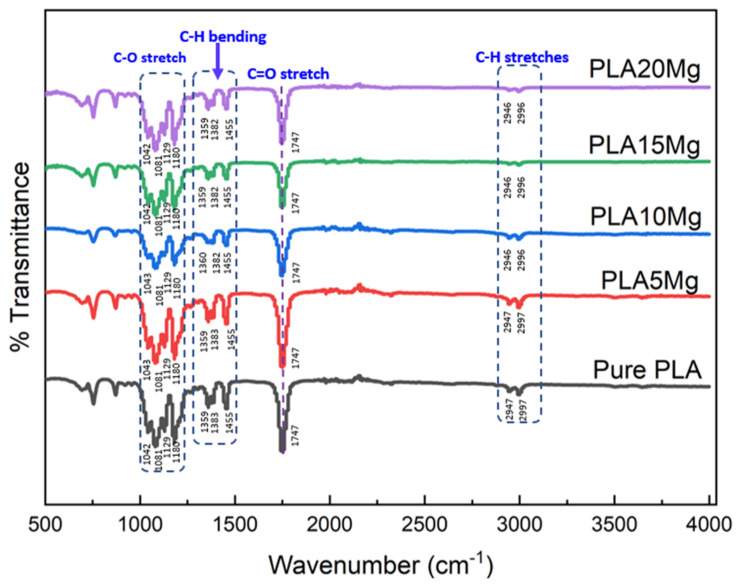
FTIR spectra of PLA and PLA/Mg composite with various Mg amounts.

To analyze a PLA/Mg composite using Fourier transform infrared (FTIR) spectroscopy, the PLA/Mg film is placed in the sample chamber of an FTIR spectrometer, and the absorption of IR radiation by the sample is measured over a range of wavelengths. FTIR spectroscopy is a technique used to identify and characterize the chemical functional groups present in a material by measuring the absorption of infrared radiation. As shown in Figure 4, the FTIR spectrum of a PLA/Mg composite shows the absorption bands of the functional groups present in the PLA matrix and the Mg-reinforcing particles. The intensity and position of the absorption bands can be used to quantify the amount of each component in the composite and to determine the degree of interaction between the two materials. The FTIR spectrum can also identify other elements present in the composite, such as additives or impurities. Only minor differences between the PLA and PLA/Mg composites suggest that the chemical linkages were comparable and compatible with the literature [30]. Even though there is a slight shift in the C-H stretch, overall, there is no significant difference in the peak positions, while the peak intensities reduced a bit after incorporating Mg particles. The intensity of the peaks at 2947 cm^−1^, associated with CH_2_ stretching, reduced as the proportion of Mg in the composite increased, which supports the idea that there is an interaction between the ester group (R-COOR’) in PLA and Mg particles. Furthermore, the peak at 1747 cm^−1^, which is a characteristic peak of PLA and is associated with the carboxyl bond of the ester group, becomes less intense as the amount of Mg in the composite increases, which indicates that the composites are undergoing degradation or an interaction with another component.

### 3.5. Scanning Electron Microscope (SEM)

**Figure 5:**
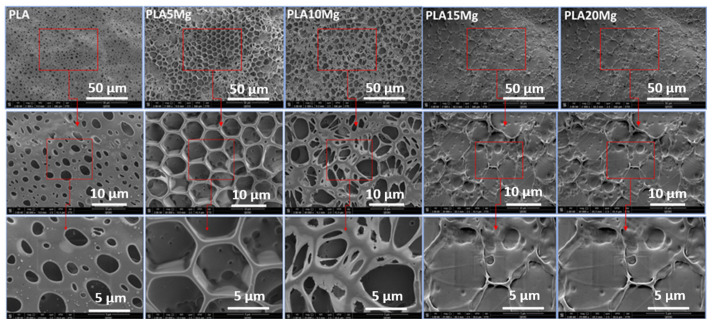
SEM images of PLA and PLA/Mg composite with various Mg amounts.

Figure 5 represents the SEM images of the PLA/Mg composite with different Mg concentrations. A PLA/Mg solution in chloroform was deposited onto a metal substrate and allowed to dry completely. The film was then removed from the substrate. The pore size and shape changed by changing the Mg particle concentration in the PLA solution. As shown in Figure 5, a porous structure is formed for pure PLA, which changes to a uniform honeycomb structure by adding 5% Mg into the solution. Various methods can be used to create a honeycomb structure in PLA/Mg composite films, including extrusion, melt-blending, and solvent casting. It is challenging to develop ordered microporous films from pure hydrophobic PLA [31,32,33,34]. However, adding certain additives to the PLA solution or the copolymerization of PLA units with other monomers can allow for the formation of honeycomb films. Mg and its alloys are excellent materials for biomedical applications due to their exceptional biodegradation and mechanical integrity [35]. In this method, we added different amounts of Mg into the PLA solution. After complete mixing, the solution was poured onto a metal plate and left to dry until the solvent had completely evaporated. We have not used any water assistance for the honeycomb structure formation in this method, as reported in the breath figure method [36,37]. A highly organized honeycomb pattern with regularly spaced uniform-sized pores was observed by adding a 5% Mg concentration into the PLA chloroform solution. As shown in the SEM images, the film exhibits a long-range hexagonal order. When the Mg particle content was increased beyond 5% (i.e., 10%), the structure deformed and the pore size increased from ~5 µm to ~8 µm, while a non-porous structure was formed by adding Mg particles beyond a 10% concentration (i.e., 15 and 20% Mg). The honeycomb structure in PLA/Mg composite films can provide several benefits, including improved mechanical properties, increased strength and stiffness, and reduced weight. The honeycomb structure can also help to distribute stress more evenly throughout the film, which can help to improve its overall durability and lifespan.

### 3.6. Degradation of PLA/Mg Composites

**Figure 6:**
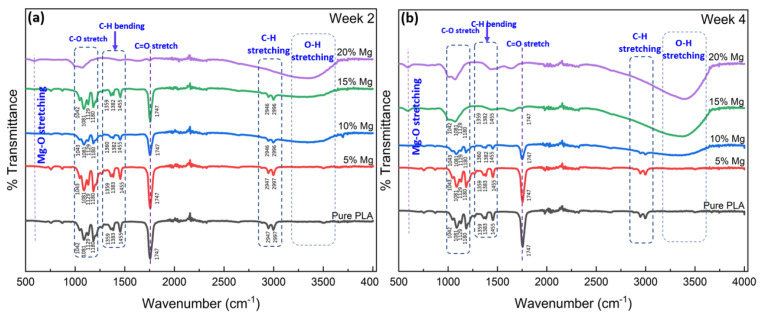
FTIR results showing the degradation of PLA and PLA/Mg composites in PBS solution dissolved for (**a**) 2 weeks and (**b**) 4 weeks.

To assess how the Mg content in PLA impacts the degradation of composites, degradation tests were conducted in vitro using a phosphate-buffered saline solution (PBS). The PLA and PLA/Mg composites were immersed in PBS for two and four weeks and then characterized via FTIR. The FTIR measurement of the samples was conducted to compare and examine the degradation caused by their immersion in PBS. Figure 6 illustrates the FTIR spectra of the films with 5, 10, 15, and 20 wt.% of Mg particles in PLA. The wide band with considerable width that appeared in the degraded films at 3400 cm^−1^ in the composites with an Mg composition of 10 wt% and beyond, attributed to the carboxylic acid group, indicates the significant degradation of the polymer chain during the composite immersion, which is significantly higher with a higher percentage of Mg in the PLA. The peak at 3000 cm^−1^, related to the stretching of the OH group caused by PLA hydrolysis, showed a similar trend and completely disappeared for the samples with 15 and 20 wt% Mg after two weeks of immersion in the PBS, as shown in Figure 6a,b. The most characteristic band of the PLA at 1747 cm^−1^ has a significantly lower intensity for the degraded composite [38,39]. As seen for both week 2 and week 4, the peak completely disappeared for the samples with 15 and 20 wt% Mg. The presence of MgO is evidenced by the small peak at 600 cm^−1^ in the 15 and 20 wt% Mg samples. The band widening at the 15 and 20 wt% Mg spectra corresponds to OH scissoring and suggest PLA degradation due to the ester bond reaction forming carboxylic acid groups. Further, the band at 1454 cm^−1^ attributed to the CH_3_ group also disappeared when the composite had 15 and 20 wt% Mg after two weeks of immersion in PBS. Additionally, an Mg-O band appeared for the samples at 590 cm^−1^. In conclusion, the Mg addition to PLA has demonstrated a faster degradation of PLA with 15 and 20 wt% Mg, while 5 wt% Mg did not affect the degradation rate of PLA much.

### 3.7. PLA/Mg Mass Variation (Weight Gain/Loss)

To study the weight loss/gain data of PLA and PLA/Mg composite films, the samples were immersed in PBS for four weeks. The samples were weighed before and after immersion for four weeks in PBS. The weight gain was calculated by measuring the sample after its removal from the PBS. These samples were dried with paper and then measured. For weight loss, the samples were oven-dried at 60 °C overnight and then measured. The weight gain (W_G_) and weight loss (W_L_) were calculated using the following equations:

W_G_ = ((W_f_ − W_0_)/W_0_) × 100 (For weight gain)

W_L_ = ((W_0_ − W_f_)/W_0_) × 100 (For weight loss)

As shown in Table 4, the sample with a high amount of Mg and a high intake weight is retained after removal from the PBS solution. Similarly, after drying the sample, the weight loss is higher for the sample with a high amount of Mg (7.7% for PLA20Mg vs. 0.1% for pure PLA). This supports the idea that Mg incorporation to PLA improves the degradation of PLA [15].

The pH of the PBS solution after four weeks was also measured and is reported in Table 4. The initial pH of the PBS measure was 7.6, which increased to 7.9 for the pure PLA after week 4 and up to 8.9 for the PLA/Mg composite with 20% Mg. The rise in the pH is due to the hydroxides produced due to Mg degradation.

### 3.8. Cell Adhesion

**Figure 7:**
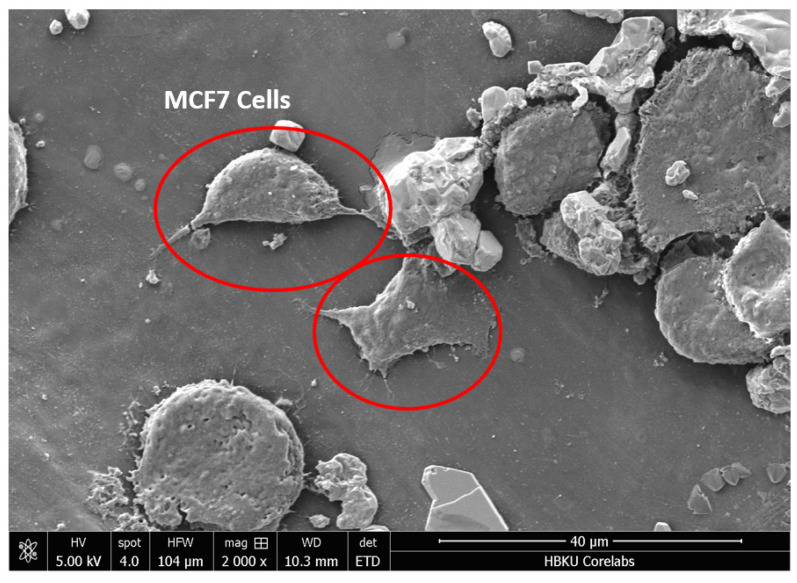
MCF7 cells attachment to PLA/10Mg composite film are encircled.

To study the biocompatibility of the PLA/Mg composite film, MCF7 cells were cultured on the PLA/10Mg film. As depicted in Figure 7, the cells were attached to the surface of PLA/Mg composite with 10% Mg. In this part of the study, we have used just one composition of the PLA/Mg composite to validate the biocompatibility of the composite film. As shown, no adverse effects were seen on the cell attachment on the PLA/Mg composite films. It is reported that various factors affect the cell adhesion to the PLA surface, including surface roughness and hydrophilicity [40]. Furthermore, the addition of Mg to PLA improves the hydrophilicity of PLA, which means the Mg content will have a positive impact on the cell adhesion and biocompatibility of the PLA/Mg composite [41].

### 3.9. Direct Ink Writing (3D Printing) of PLA/Mg Ink

**Figure 8:**
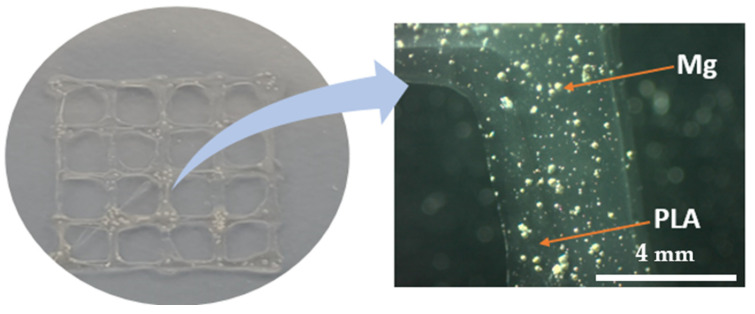
3D-printed mesh structure using bioprinting process.

The ink’s rheological properties refer to its flow behavior and mechanical properties, such as its viscosity, elasticity, and shear-thinning behavior, and printing parameters are the most critical parameters for a successful printing process. These properties can impact the extrusion of the bioink from the printing nozzle, as well as the stability and shape retention of the printed structure. The bioink must have the appropriate rheological properties to allow for the precise deposition and layer-by-layer building of the structure. After several experimental trials, a viscosity of 10^6^ was found to be optimum for a successful bioprinting process. Besides rheological properties, an optimal set of 3DP process parameters is vital to achieve the desired shape stability. Therefore, extensive experimentation to achieve a successful 3D print was performed, and with a printing speed of 2 mm/s and a layer height of 0.1 mm, the designed geometry was successfully fabricated. It is worth noting that the optimal set of process parameters varies based on the material compositions and may require optimization for different Mg content in the PLA matrix. Therefore, using the optimal process parameters, the designed mesh was successfully fabricated using PLA/Mg composites with 5 wt% of Mg content. The 3D-printed structure was manufactured with appropriate dimensional accuracy, as reported in Figure 8. The uniform distribution of Mg particles within the PLA matrix is also crucial to achieving the desired performance of 3D-printed composites. Therefore, the 3D-printed mesh was examined under a microscope, and homogenously dispersed Mg particles within the extrudate were observed, as confirmed by the microscopic images. This study presents a preliminary analysis of the potential of using PLA/Mg composite ink using DIW 3D printing to fabricate 3D structures. However, future studies will report complex scaffold shapes, the optimization of other PLA/Mg composite compositions, and the optimization of the bioprinting process.

## 4. Conclusions

In this study, we successfully synthesized a honeycomb surface pattern using a PLA/Mg composite with a relatively simple solution casting technique without any complicated water droplet technique. The effect of the Mg percentage on the PLA/Mg composite surface morphology, thermal behavior, and degradation was studied. Incorporating Mg particles played an essential role in defining the structural morphology and enhancing the degradation rate of the PLA/Mg composite films. A well-organized honeycomb structure was obtained by adding a 5% Mg concentration to the PLA, which deformed into a porous structure by increasing the Mg amount to 10% and onwards. The FTIR results showed a significant increase in the degradation of the PLA with 15 and 20 wt% of Mg particles. Furthermore, the PLA/Mg ink was successfully 3D-printed with the DIW printer. DIW 3D printing allows for precise control of the amount and placement of the ink and can be used to create complex structures or designs without making the filaments. Optimal ink viscosity is crucial for achieving higher print quality. Therefore, conducting further rheological studies is necessary to enhance ink properties for improved printability. DIW 3D printing has the potential to revolutionize tissue engineering and regenerative medicine by enabling the creation of custom tissues and organs for patients in need. However, the technology is still in its early stages, and more research is needed to optimize the ink materials, printing parameters, and post-printing processing techniques to achieve the desired outcomes.

## Figures and Tables

**Table 1:** Compositions of synthesized PLA/Mg composites.

Material	Polymer Matrix (w%)	
	PLA	Mg
PLA	100	0
PLA/5Mg	95	5
PLA/10Mg	90	10
PLA/15Mg	85	15
PLA/20Mg	80	20

**Table 2:** Details of the mesh design and 3DP process parameters.

Mesh Design Specifications	
Overall Dimensions	12.4 mm × 12.4 mm × 0.4 mm
Strut Thickness	0.4 mm
Strut Spacing	3 mm
**3DP Process Parameters**	
Printing Speed	2 mm/s
Layer Height	0.1 mm
Infill Density	100%

**Table 3:** Thermal properties of PLA/Mg composite films with varying Mg content.

Materials	T_g_	T_m_	T_d_
PLA	41	167	340
PLA/5Mg	48	172	295
PLA/10Mg	50	173	295
PLA/15Mg	47	170	290
PLA/20Mg	49	172	282

**Table 4:** Mass variation of PLA and PLA/Mg films after four weeks of immersion in PBS solution, pH values, and weight gain/loss data.

Materials	pH Values	Weight Gain/Loss	
		Weight Gain (%)	Weight Loss (%)
PLA	7.9	0.9	0.1
5%Mg	8.6	1.0	1.5
10%Mg	8.8	3.0	5.0
15%Mg	8.85	5.0	7.0
20%Mg	8.89	5.2	7.7

## Data Availability

All data generated or analyzed during this study are included in this article.
